# Foreign language reading and spelling in gifted students with dyslexia in secondary education

**DOI:** 10.1007/s11145-016-9717-x

**Published:** 2017-01-09

**Authors:** Sietske van Viersen, Elise H. de Bree, Lilian Kalee, Evelyn H. Kroesbergen, Peter F. de Jong

**Affiliations:** 1 Research Institute of Child Development and Education (RICDE), University of Amsterdam, PO Box 15780, 1001 NG Amsterdam, The Netherlands; 2 Department of Pedagogical and Educational Sciences, Utrecht University, PO Box 80140, 3508 TC Utrecht, The Netherlands

**Keywords:** Dyslexia, Giftedness, Foreign language, Secondary education, Bayes

## Abstract

A few studies suggest that gifted children with dyslexia have better literacy skills than averagely intelligent children with dyslexia. This finding aligns with the hypothesis that giftedness-related factors provide compensation for poor reading. The present study investigated whether, as in the native language (NL), the level of foreign language (FL) literacy of gifted students with dyslexia is higher than the literacy level of averagely intelligent students with dyslexia and whether this difference can be accounted for by the difference in their NL literacy level. The sample consisted of 148 Dutch native speaking secondary school students divided in four groups: dyslexia, gifted/dyslexia, typically developing (TD), and gifted. All students were assessed on word reading and orthographic knowledge in Dutch and English when they were in 7th or 8th grade. A subsample (*n* = 71) was (re)assessed on Dutch, English, French, and German literacy one year later. Results showed that Dutch gifted students with dyslexia have higher NL literacy levels than averagely intelligent students with dyslexia. As in the NL, a stepwise pattern of group differences was found for English word reading and spelling, i.e., dyslexia < gifted/dyslexia < TD < gifted. However, it was not found for French and German literacy performance. These results point towards compensation: the higher English literacy levels of gifted/dyslexic students compared to their averagely intelligent dyslexic peers result from mechanisms that are unique to English as a FL. Differences in results between FLs are discussed in terms of variation in orthographic transparency and exposure.

## Introduction

A few studies have suggested that gifted children with dyslexia, despite having severe and persistent word level literacy difficulties, read and spell better than their averagely intelligent peers with dyslexia (Berninger & Abbott, 2013; van Viersen, Kroesbergen, Slot, & de Bree, 2016). These studies have mainly concerned primary school children’s literacy levels in the native language (NL). The current study focused on secondary education students’ reading and spelling acquisition in a foreign language (FL). We examined whether, as in the NL, the level of FL literacy of gifted students with dyslexia is higher than the literacy level of averagely intelligent students with dyslexia. In addition, we investigated whether such a difference can be accounted for by the difference in literacy level in their NL.

There are two possible explanations for the attested higher literacy level of gifted children with dyslexia compared to averagely intelligent children with dyslexia. The first is that these children have a less severe underlying (cognitive) deficit. In other words, gifted children with dyslexia have somewhat higher literacy levels simply because they have slightly better literacy-related skills (core-deficit account; Stanovich & Siegel, 1994). Such findings have indeed been reported (van Viersen, de Bree, Kroesbergen, Slot, & de Jong, 2015). However, van Viersen et al. (2015) argue that the higher performance of gifted children with dyslexia on tasks measuring underlying deficits is not the result of better underlying skills, but is likely due to giftedness-related strengths (e.g., processing speed; Johnson, Im-Bolter, & Pascual-Leone, 2003) impacting on task-specific aspects unrelated to the targeted skill. An alternative explanation would be that gifted students with dyslexia actually have equally severe deficits but have higher literacy levels due to compensation (compensation account; e.g., Foley Nicpon, Allmon, Sieck, & Stinson, 2011). Here, compensation refers to the development of compensatory mechanisms or strategies associated with specific protective factors that are relatively unrelated to the underlying deficit of dyslexia but known to influence literacy (e.g., orthographic compensation, see below; van der Leij & van Daal, 1999). These compensatory mechanisms could be used to circumvent an underlying deficit or subdue its negative effect and thereby improve literacy performance (see also van Viersen et al., 2015). Unfortunately, both accounts are hard to separate based on descriptive data in the NL. As we will argue below, investigating both dyslexic groups on their FL skills may provide support for the compensation account and disentangle it from the core-deficit account.

A comparison between the FL literacy outcomes of gifted students with dyslexia and averagely intelligent students with dyslexia could yield similar findings as in the NL. According to the Linguistic Coding Differences (LCD) hypothesis (Sparks & Ganschow, 1991, 1993, 1995) NL skills are fundamental for the acquisition of a FL. A large body of empirical research found strong associations between NL and FL literacy performance (e.g., Morfidi, van der Leij, de Jong, Scheltinga, & Bekebrede, 2007; Sparks, Patton, Ganschow, Humbach, & Javorsky, 2006). To the best of our knowledge, only one study has demonstrated that this association also holds for students with dyslexia. Morfidi et al. (2007) found lower FL performance for poor readers compared to average readers, specifically in word reading and orthographic knowledge. Moreover, they showed that NL word reading is the strongest predictor of FL word reading performance after controlling for age, vocabulary, and reading group (Morfidi et al., 2007). Accordingly, the LCD hypothesis seems to provide a logic behind the difficulties that students with dyslexia experience in FLs.

However, it is uncertain whether the LCD hypothesis could also explain the differences in literacy level between dyslexic students with and without giftedness, especially when considering their different underlying cognitive profiles (Berninger & Abbot, 2013; van Viersen et al., 2016). The main assumption of the LCD hypothesis is that the association between NL and FL performance is strong because the same underlying factors are involved in the NL and FL (e.g., Sparks, 1995). Indeed, several studies have shown that underlying cognitive skills, such as word decoding-related phonological and orthographic processing, but also vocabulary and grammar, contribute to both NL and FL learning (e.g., Dufva & Voeten, 1999; Kahn-Horwitz, Shimron, & Sparks, 2006; Lindsey, Manis, & Bailey, 2003; Melby-Lervåg & Lervåg, 2011; Morfidi et al., 2007; see Koda, 2005 for an overview). Moreover, difficulties in NL phonology were found to surface in FL phonology (Morfidi et al., 2007), indicating cross-linguistic transfer of underlying deficits associated with dyslexia. Evidence for possible transfer of strengths in cognitive factors is limited though (but see Lindsey et al., 2000; Sparks, Patton, Ganschow, & Humbach, 2012 for some suggestions). It is thus unclear to what extent both underlying risk and protective factors of the NL overlap with factors involved in the FL and whether they are responsible for differences in literacy levels between gifted and averagely intelligent students with dyslexia.

Based on the LCD hypothesis, controlling for NL literacy skills should eliminate differences in FL literacy level between gifted and averagely intelligent students with dyslexia, as these differences are assumed to have their origin in the NL. However, several studies have indicated that (differences in) NL literacy skills do not always translate to FL literacy. Some students with dyslexia were found to read better in a FL than expected based on their NL, especially when the FL is English (e.g., Bekebrede, van der Leij, & Share, 2009; Miller-Guron & Lundberg, 2000; van der Leij & Morfidi, 2006). This better than expected performance is thought to result from a different type of reading process and has been referred to in terms of compensation. For example, higher literacy performance in the FL of students with dyslexia could be the result of an alternative reading strategy, in which the focus is on sight-word storage or larger orthographic units instead of grapheme-phoneme decoding (Bekebrede et al., 2009). Such a reading strategy fosters both reading accuracy and fluency. It is evoked by the greater irregularity of English as an opaque language, in which phonological decoding may be less effective for reading (Seymour, Aro, & Erskine, 2003; Ziegler & Goswami, 2005). Effective use of this alternative strategy, also called orthographic compensation (van der Leij & van Daal, 1999), is thought to be driven by the amount of exposure to print, which is generally larger for English than other foreign languages (see Rack, Snowling, & Olson, 1992; Stanovich & West, 1989). Bekebrede et al. (2009) have shown that secondary school students with dyslexia with higher levels of NL and FL orthographic knowledge show higher English reading performance as well as higher English spelling levels compared to dyslexic students with low orthographic competence. These results suggest that group differences in FL literacy performance would be caused by additional factors, unique to the specific FL, that alter the reading process, resulting in higher literacy levels. Accordingly, differences between gifted and averagely intelligent students with dyslexia may remain present after controlling for NL literacy. However, the results of the above-mentioned studies cannot be directly generalized to other FLs than English.

The influence of NL skills on FL performance may vary between FLs based on language-specific characteristics, such as transparency or exposure (see also Geva & Siegel, 2000). Orthographic transparency refers to (the complexity of) the letter-sound correspondences within a language, which can be consistent (e.g., German and Dutch) or (very) inconsistent (English and French; e.g., Seymour et al., 2003). As outlined above, the high orthographic complexity of English may increase the effect of processes associated with orthographic compensation. Yet, the benefit of advanced orthographic knowledge may be smaller in, for example, French than English or even absent in German. For the latter language, grapheme-phoneme decoding is straightforward and thereby less error prone than in languages that have inconsistent grapheme-phoneme correspondences. In addition, exposure to print (e.g., Brown, Waring, & Donkaewbua, 2008; Mol & Bus, 2011) and to spoken language (e.g., Bisson, van Heuven, Conklin, & Tunney, 2013) have been found to foster FL acquisition, in particular vocabulary. Knowledge of the pronunciation and meaning of words is especially important for orthographic learning in languages with many irregular words (Wang, Nickels, Nation, & Castles, 2013), such as English and French. Considering the dominance of English in today’s increasingly multilingual society, both types of exposure may be much more intensive for English than for French and German. Variation in the amount of exposure may thus partly determine the extent to which students have the opportunity to develop compensatory mechanisms based on literacy-related strengths, such as the orthographic compensation described above, or may have to rely on their NL skills. Therefore, differences between FLs in the influence of NL skills on FL literacy can be expected.

### Current study

In the present study, we compared four groups of secondary school students with and without giftedness and/or dyslexia, which resulted from the cross-classification of giftedness (yes vs. no) and dyslexia (yes vs. no). Giftedness was defined as academic giftedness or high intelligence (e.g., Winner, 1997), whereas dyslexia was defined as severe and persistent literacy difficulties (e.g., Snowling, 2000). Although the main focus is on the difference in literacy level between both dyslexic groups, typically developing (TD) and gifted groups were also included as a reference for (above) average literacy skills and to provide a more complete overview of group differences in FL performance. First, we examined whether a stepwise pattern of group differences previously found in NL reading and spelling performance (i.e., dyslexia < gifted/dyslexia < TD < gifted) is also attested in FLs. If the pattern is replicated, the second question pertains to whether the group differences, particularly between both dyslexic groups, can be accounted for by the difference in NL literacy level. Controlling for NL skills, thereby also cancelling out NL subskills and compensatory mechanisms, can reveal if differences in literacy levels have their origin in the NL, as suggested by the LCD hypothesis, or may involve factors or processes that are unique to the (specific) FL (e.g., Bekebrede et al., 2009; Morfidi et al., 2007). If NL skills cannot account for the pattern of FL performance, this would indicate that unique factors or processes are responsible for the higher literacy performance of gifted students with dyslexia in the specific FL, providing evidence for the existence of compensation.

In this study, all students had Dutch as their NL. English, French, and German were the FLs under investigation. For all three FLs, we expected to replicate the stepwise pattern previously found in the NL (as described above). Several additional hypotheses were formulated with respect to the different FLs. Specifically, the degree to which this pattern can be accounted for by NL skills was expected to differ between FLs. For English, the language with least orthographic transparency and most exposure, we hypothesized that the stepwise pattern would remain intact after controlling for NL literacy. For French, a less opaque language than English but with generally less exposure, we also hypothesized a stepwise pattern of group differences to remain intact, but possibly less clear than for English. German, however, is a more transparent language than Dutch and comparable to French in terms of exposure. We hypothesized that the great similarity to Dutch would either facilitate or hamper FL performance, which may result in comparable FL levels between both dyslexic groups.

## Method

### Participants

The total sample consisted of 148 Dutch students in secondary education. Parents and students were recruited through calls on the websites of educational magazines, blogs, and contacts with school teachers and clinicians. Written informed consent was obtained from all parents and students. It is important to note that Dutch secondary education is highly tracked (see SBB, 2015). After 6th grade, students continue education at the level befitting their prior development in primary education. A rough distinction can be made between pre-vocational education (vmbo; 4 years), higher pre-vocational education (havo; 5 years), and pre-university education (vwo; 6 years), of which the upper and lower tracks can also be divided into hierarchical sub-tracks. Generally, students start secondary education with two years in a combined track class (e.g., havo/vwo) and then continue with the track that fits best. As the level and amount of instruction may differ between tracks and students came from a variety of (sub-)tracks to cover all four research groups (see Table 1), this needs to be controlled for in the analyses (see below).

**Table 1:** Background characteristics of the four groups at the two time points

Measure	T1				T2			
	D	GD	TD	G	D	GD	TD	G
*n*	32	19	39	36	16	15	16	24
Boys (%)	40.6	78.9	43.6	47.2	25.0	73.3	56.3	62.5
Dyslexia (%)^a^	68.8	100.0	0.0	0.0	81.3	100.0	0.0	0.0
Gifted (%)^a^	0.0	63.2	0.0	36.1	0.0	53.3	0.0	37.5
Age (months)	154.91_a_ (7.99)	156.42_a_ (7.16)	157.10_a_ (7.03)	154.28_a_ (9.11)	168.81_a_ (8.48)	170.13_a_ (7.14)	170.81_a_ (6.58)	167.42_a_ (8.91)
Level of education^b^	5.47_a_ (1.83)	6.89_b_ (1.49)	6.31_ab_ (2.27)	8.11_c_ (2.04)	5.19_a_ (1.80)	6.53_a_ (2.07)	6.06_a_ (2.14)	8.50_b_ (1.98)
IQ (total)	102.91_a_ (8.82)	129.63_b_ (7.71)	106.23_a_ (8.99)	132.89_b_ (8.92)	102.00_a_ (9.75)	130.53_b_ (6.71)	106.25_a_ (9.23)	133.75_b_ (8.80)
Dutch word reading^c^	5.28_a_ (3.14)	6.37_a_ (3.10)	11.67_b_ (2.20)	12.81_b_ (2.45)	6.50_a_ (4.23)	7.80_a_ (3.26)	11.12_b_ (1.71)	12.88_b_ (2.25)
Dutch pseudo-word reading^c^	5.36_a_ (2.25)	5.26_a_ (2.64)	11.38_b_ (2.55)	12.56_c_ (9.27)	–	–	–	–
Dutch spelling^d^	1.69_a_ (1.00)	2.68_b_ (1.86)	5.21_c_ (1.70)	6.78_d_ (1.87)	–	–	–	–

The group means are given with the standard deviations in parentheses. Means in the same row per time point that do not share subscripts differ at *p* < .05

*D* dyslexia, *GD* gifted + dyslexia, *TD* typically developing, *G* gifted

^a^Official diagnosis provided by a professional

^b^On a scale from 1 to 10, lowest track (basic pre-vocational education) representing 1, highest track (bilingual pre-university education) representing 10

^c^Standard score with a mean of 10 and standard deviation of 3

^d^Stanine score on a scale from 1 to 9

The sample was divided into groups based on definitions of giftedness and dyslexia. For giftedness, the cut-off value was set at a full IQ score >120 or a 95% reliability interval around a score of 125 (i.e., 116–131) in case of a short form. For dyslexia, students had to show 1) reading scores below the 10th percentile (standard score ≤ 6), or 2) reading scores below the 15th percentile (standard score ≤ 7) *and* spelling scores below the 10th percentile (stanine ≤ 2), which is in line with Dutch protocols for diagnosing dyslexia (e.g., Kleijnen et al., 2008). Of all students with an official dyslexia diagnosis, 22 gifted students were excluded from further analyses in this study because their reading and/or spelling levels no longer fell below the diagnostic threshold for dyslexia described above. TD students had to show reading and spelling scores above the 25th percentile. This resulted in four research groups covering students with dyslexia (*n* = 32), gifted students with dyslexia (*n* = 19), TD students (*n* = 39), and gifted students (*n* = 36). All students within both dyslexia groups had below average scores (standard score < 8) on at least one out of three measures for phonological processing (i.e., phonological awareness, rapid automatized naming, or verbal short-term memory).

It is important to note that in The Netherlands it is common for children to grow up learning one language during the first years of primary school. FL education (mostly English) has recently been introduced at the end of primary education (Grades 5 and 6). At the time of data collection, it was only a mandatory part of the curriculum from the start of secondary education (Grade 7 onwards). All students start with two FLs in 7th grade (i.e., English and either French or German). A third language is added in 8th grade. Therefore, data was collected at two points during secondary education. The first measurement took place when students were in 7th or 8th grade and concerned a broad screening of NL and FL literacy and literacy-related skills. For literacy, the focus was on Dutch and English, because most students had a sufficient basis in both languages. The second measurement comprised an additional assessment of a subsample of 71 students one year later (see Procedure), when they were in 8th or 9th grade. These students were selected based on willingness to participate and kept the group classification of their initial assessment one year earlier. Besides reassessment of Dutch and English literacy, the main focus was on French and German literacy, in which most students received at least several months of formal instruction by this time. Background characteristics of the samples at both occasions are displayed in Table 1.

### Instruments

#### Intelligence

The general cognitive abilities of the students were assessed using the Dutch version (Kort et al., 2005) of the *Wechsler Intelligence Scale for Children*—*Third Edition* (WISC-III; Wechsler, 1991). If recent test results were available (i.e., not older than two years), students were not reassessed. If not, a short form was used, consisting of two verbal subtests (i.e., vocabulary and similarities) and two performance subtests (i.e., block design and picture arrangement). Total IQ-scores could be computed based on the sum of the standardized subtest scores (see Kaufman, Kaufman, Balgopal, & McLean, 1996). Reliability and validity quotients of the short form are all greater than .83 (Kaufman et al., 1996).

#### Word reading

Timed word reading ability was assessed in four languages. For Dutch, the *Eén*-*Minuut*-*Test* (EMT; Brus & Voeten, 1999) was used. The *TOWRE* sight word efficiency subtest (Torgesen, Wagner, & Rashotte, 1999) was used for English. The French task was part of a screening tool for dyslexia in secondary education (Kleijnen, Steenbeek-Planting, & Verhoeven, 2008). For German, an experimental task was composed from a large list of words that are generally taught in the first two grades of secondary education. Word length increased from one to four syllables on all tasks. The student had one min to accurately read aloud as many words as possible per task. Maximum scores are 116, 108, 100, and 100, respectively. On all tasks, raw scores were the number of correctly read words within the allotted time. Internal consistency of the Dutch task is .90 (Evers et al., 2009–2012). For the English task it ranged between .93 and .96 (Torgesen et al., 1999).

In Dutch, timed pseudoword reading was measured using *Klepel* (van den Bos, lutje Spelberg, Scheepstra, & de Vries, 1994). The student had two min to accurately read as many pseudowords as possible. Here, word length also increased from one to four syllables. Raw scores were the number of correctly read pseudowords, with a maximum of 116 words. Internal consistency is .92 (Evers et al., 2009–2012). The Dutch word reading and pseudoword reading tasks were used for the inclusion criteria of dyslexia, for which raw scores were transformed into norm-referenced standard scores (*M* = 10, *SD* = 3).

#### Orthographic knowledge

Production of orthographic knowledge in Dutch and English was assessed using two spelling measures. For Dutch, a normed sentence dictation from the dyslexia protocol for secondary education (Henneman & Kleijnen, 2005) was used. This spelling test, which is about the Dutch weather, consists of 10 sentences of increasing length and difficulty. The total number of correctly spelled words was the raw score, which was used in the analyses. Raw scores were transformed into norm-referenced stanine scores for application of the criteria for dyslexia. For English, an experimental spelling task was used (S. van Viersen & E. H. de Bree, personal communication, October 2014). The task consisted of three blocks of 10 sentences that provided context to the target words. Children had to write down the target word after the sentence was read. The target words were selected from the listed words in the English word reading task. The raw score was the total number of correctly spelled words.

In English, French, and German, orthographic knowledge was (also) assessed using orthographic choice tasks, which were aimed at recognition instead of production. For French and German this type of task was used as an alternative to a dictation, which was thought to be too difficult at this stage of development for most students. The English orthographic choice task (Olsen, Forsberg, Wise, & Rack, 1994) was used as a model for developing the experimental French and German tasks, for which items were composed by selecting words from the respective word reading tasks. Students received a list of 40 items (one list per language) consisting of correctly spelled meaningful words and incorrectly spelled words with the same pronunciation (e.g., English: wurd–word). Students had to recognize and select the word with the correct spelling. Word length increased from one to four syllables. The raw score was the total number of correctly recognized words, which was used in the analyses. Internal consistency (Cronbach’s α) of the English task was .78 (Bekebrede et al., 2009).

### Procedure

Assessments were conducted by trained and supervised undergraduate and graduate students. Their training comprised an intensive instructional session about the test battery and a test session with a student, on which they received extensive feedback. Students were first tested in 7th or 8th grade, during one two- to three-h session (with ample breaks provided) at home, school, or in a clinic. The first assessment covered intelligence as well as all tasks for Dutch and English language reported in this study. One year later (in 8th or 9th grade), a subsample was assessed again during a 30- to 40-min session covering also the French and German tasks. During both assessments, tasks were always presented in a fixed order. For the order of the languages, the least familiar languages were assessed first (i.e., T2: French and German) as they might take more effort, followed by the more familiar languages (i.e., T1 and T2: English and Dutch). Languages were always separated by intermediate tasks to prevent interference between languages. Furthermore, within languages the order of the tasks was also fixed (i.e., spelling, orthographic choice, word reading) to minimize carry over effects across tasks targeting the same language.

### Analyses

Bayesian analyses were used to compare groups on their word reading and orthographic knowledge in the four languages. This approach was favored over more traditional methods for several reasons. First, Bayesian model selection (i.e., the Bayesian alternative to traditional frequentist ANOVA) allows for the testing of equality or inequality constrained informative hypotheses, which can be formulated using prior knowledge (Klugkist, Laudy, & Hoijtink, 2005). It is also possible to compare several competing informative hypotheses that represent opposing views or specific expectations. Second, it is suitable for dealing with non-normal data, since Bayesian analyses are not based on normality or asymptotic assumptions (Gill, 2008). As such, it is also a good alternative when having to work with small samples or unequal group sizes. Third, these specific analyses do not involve traditional *p*-values, thereby avoiding multiple testing problems such as alpha inflation or loss of power when adopting a stricter alpha level (Klugkist, van Wesel, & Bullens, 2011).

In short, the hypothesized differences between group means on word reading and orthographic knowledge were translated into equality and inequality constrained statistical hypotheses (Table 2; see also van Viersen et al., 2016). These informative hypotheses were each compared to the alternative hypothesis (i.e., empty model without constraints). Each comparison results in a Bayes factor (BF), representing the amount of evidence in favor of one hypothesis compared to another (Kass & Raftery, 1995). BFs > 1 indicate support for the informative hypothesis, whereas BFs < 1 indicate support for the alternative hypothesis. The BF can also be interpreted as a measure of effect size (i.e., BF 1–3 = small, BF 3–10 = medium, BF > 10 = large; Kass & Raftery, 1995). Competing hypotheses can be compared based on the posterior model probability (PMP), representing the relative support for a specific hypothesis within a set of hypotheses (Klugkist et al., 2011). In general, a difference between PMPs that is smaller than .05 cannot be interpreted and results in equal support for both models. A more detailed description of Bayesian model selection within this field of research is provided by van Viersen et al. (2015).

**Table 2:** Statistical hypotheses Bayesian model selection

Hypothesis	Model	Statistical notation
Alternative	Model 0	μ_D_, μ_GD_, μ_TD_, μ_G_
Informative 1	Model 1	μ_D_ = μ_GD_ < μ_TD_ < μ_G_
Informative 2	Model 2	μ_D_ < μ_GD_ < μ_TD_ < μ_G_

*μ* group mean, *D* dyslexia, *GD* gifted + dyslexia, *TD* typically developing, *G* gifted

Several covariates were used in the analyses. Group differences were controlled for two types of exposure, i.e., educational level (track, as a proxy for level of instruction) and language-specific didactical age (quantity, number of months a student has received formal education in a specific FL, with a maximum of 10 months per year), at both time points. Controlling for NL skills in the second part of the analyses was done by including Dutch word reading or spelling as (separate) covariates for word reading and orthographic knowledge in English, French, and German. The analyses were conducted using the BIEMS software package (Mulder, Hoijtink, & Klugkist, 2010; Mulder, Hoijtink, & de Leeuw, 2012; Mulder et al., 2009).

## Results

Missing data analysis revealed no missing data points. An outlier analysis using z-scores revealed two outliers (*z*-score < −3.29) on two different dependent variables. These outliers were corrected to the closest raw score that corresponded to a *z*-score >−3.29. Checking the assumptions for ANCOVA revealed that four dependent variables (i.e., Dutch and English spelling at T1 and French and German orthographic knowledge at T2) and all covariates were not normally distributed. Hence, Bayesian model selection was considered an appropriate alternative to compare groups.

### NL profile

Patterns of group differences were first investigated in the NL to establish that the stepwise pattern previously found in primary education (van Viersen et al., 2016) is also attested in early secondary education (i.e., T1). Table 3 displays the posterior means and standard deviations of the four groups and the BFs and PMPs for the three models under investigation. The results show that for both word reading and spelling Model 2 received most support from the data (PMPs .54 and .88), with BFs indicating strong effects. The posterior means of the unconstrained model indeed indicate a stepwise pattern, as gifted students with dyslexia scored in between students with dyslexia and TD students, and gifted students outperformed all groups. Dutch word reading patterns of group differences are comparable across both occasions of measurements (see Table 3).

**Table 3:** Posterior means (PM) and standard deviations (PSD) of the literacy measures for the four languages adjusted for educational level and FL-specific didactical age and Bayes factors (BF) and posterior model probabilities (PMP) of the three models under investigation

Skill	Dyslexia		Gifted + dyslexia		Typically developing		Gifted		Model 0		Model 1		Model 2	
	PM	PSD	PM	PSD	PM	PSD	PM	PSD	BF	PMP	BF	PMP	BF	PMP
T1														
Dutch														
Word reading	63.04	4.33	66.23	6.48	88.49	3.28	90.95	3.99	1.00	.03	13.21	.43	**16.54**	**.54**
Spelling	149.91	1.95	154.79	2.96	164.70	1.50	167.29	1.87	1.00	.04	1.95	.08	**21.52**	**.88**
English														
Word reading	51.03	2.66	55.49	3.75	63.16	1.96	68.36	2.38	1.00	.03	4.63	.16	**23.15**	**.80**
Orthographic choice	33.42	0.23	33.65	0.33	35.54	0.17	37.18	0.21	1.00	.03	**19.70**	**.56**	14.58	.41
Spelling	13.31	0.45	15.66	0.63	18.20	0.33	20.45	0.40	1.00	.04	1.84	.07	**22.84**	**.89**
T2														
Dutch														
Word reading	69.87	11.36	74.03	10.56	86.31	10.17	94.20	8.37	1.00	.03	11.72	.37	**19.18**	**.60**
English														
Word reading	58.26	6.11	63.65	5.58	71.03	5.39	78.36	4.43	1.00	.03	5.18	.18	**22.65**	**.79**
French														
Word reading	26.65	10.94	28.74	10.40	37.37	9.85	43.21	8.18	1.00	.03	**13.61**	**.47**	**14.54**	**.50**
Orthographic choice	33.33	0.49	33.22	0.47	34.51	0.45	36.70	0.37	1.00	.04	**14.79**	**.59**	9.35	.37
German														
Word reading	29.61	7.10	31.19	6.92	40.29	6.39	43.75	5.31	1.00	.04	**12.93**	**.47**	**13.46**	**.49**
Orthographic choice	26.88	1.04	23.38	1.04	26.56	0.95	27.18	0.78	**1.00**	**.66**	0.42	.28	0.10	.07

Bold values indicate BFs and PMPs of models that received most support from the data

### FL profiles

The group comparisons on FL reading and orthographic knowledge show considerable differences between languages and literacy skills (see Table 3). The pattern for English is largely the same as for Dutch. The BF of Model 2 indicates that the model received about 23 times more support from the data than the alternative model, indicating strong effects for both word reading^1^ and spelling (PMPs .80 and .89). Yet, for orthographic choice Model 1 received most support from the data (PMP = .56), also indicating a large effect. The posterior means confirm that students with dyslexia and gifted students with dyslexia obtained about equal orthographic choice scores. The results also indicate comparable patterns of group differences for English word reading at the two measurement occasions.

For word reading^2^ in French Model 1 and 2 received a comparable amount of support from the data. This was about 14 times more than the alternative model, which can be considered a large effect. Based on the PMPs (.47 and .50) neither of the models can be preferred over the other. For French orthographic choice, Model 1 received most support from the data, also about 14 times more than for the alternative model. Overall, the posterior means indicate there are no clearly identifiable differences between averagely intelligent students with dyslexia and gifted students with dyslexia in French reading and orthographic knowledge.

The pattern for German word reading is about the same as for French word reading. Model 1 and 2 both received about 13 times more support from the data than the alternative model (PMPs .47 and .49). There is no clear difference between averagely intelligent students with dyslexia and gifted students with dyslexia. However, TD students still obtained higher scores and gifted students outperformed all groups. For orthographic choice, the pattern is not in line with the expectations, as the alternative model received most support from the data (PMP = .66). The posterior means show that the averagely intelligent students with dyslexia, the TD students and the gifted students all showed comparable performance, but the gifted students with dyslexia obtained considerably lower scores (Table 3).

### FL profiles when controlling for NL

In order to gain more insight in the influence of NL skills on FL performance, the group differences in English, French, and German reading, spelling and orthographic choice were controlled for word reading and spelling proficiency in Dutch. Overall, controlling for NL skills resulted in smaller effect sizes and less clear group differences (see Table 4)—indicating an influence of NL on FL performance. Yet, the stepwise pattern for the English measures remained intact (both at T1 and T2), indicating that the higher performance of gifted students with dyslexia is the result of factors/mechanisms that are unique to English as a FL. Model 2 received most support from the data for both word reading and spelling (PMPs .54 and .78). The posterior means indicate that the word reading ability of the gifted students with dyslexia moved more towards that of the TD students (even slightly above), while averagely intelligent students with dyslexia still had the lowest and gifted students the highest scores. This shift in pattern is illustrated by the considerably lower, but still medium-sized, BF for word reading. The same trend was visible for group differences in spelling. The difference between the gifted students with dyslexia and TD students has become notably smaller. However, gifted students with dyslexia did not close the gap entirely, which kept the stepwise pattern intact as indicated by the larger effect size for English spelling than for word reading. For English orthographic choice the results were unchanged. Model 1 still received most support from the data (PMP = .64). The posterior means show the same pattern, indicating about equal performance for averagely intelligent students with dyslexia and gifted students with dyslexia.

**Table 4:** Posterior means (PM) and standard deviations (PSD) of the literacy measures for the three foreign languages adjusted for educational level, FL-specific didactical age, and Dutch reading or spelling and Bayes factors (BF) and posterior model probabilities (PMP) of the three models under investigation

Skill	Dyslexia		Gifted + Dyslexia		Typically Developing		Gifted		Model 0		Model 1		Model 2	
	PM	PSD	PM	PSD	PM	PSD	PM	PSD	BF	PMP	BF	PMP	BF	PMP
T1														
English														
Word reading^a^	57.70	2.65	60.77	3.19	59.50	1.69	63.61	2.08	1.00	.10	3.72	.36	**5.54**	**.54**
Orthographic choice^b^	34.27	0.29	34.16	0.33	35.18	0.17	36.55	0.23	1.00	.04	**16.33**	**.62**	8.83	.34
Spelling^b^	14.80	0.55	16.52	0.62	17.56	0.33	19.35	0.43	1.00	.04	4.38	.18	**19.21**	**.78**
T2														
English														
Word reading^a^	64.27	4.90	67.44	4.18	69.44	3.81	73.04	3.70	1.00	.05	6.22	.29	**14.13**	**.66**
French														
Word reading^a^	32.77	10.84	33.64	9.86	34.63	8.63	37.94	8.31	1.00	.08	**6.82**	**.55**	4.62	.37
Orthographic choice^b^	34.29	0.65	33.60	0.46	34.22	0.44	36.03	0.43	1.00	.09	**7.13**	**.64**	3.09	.28
German														
Word reading^a^	36.30	5.75	36.35	5.31	36.91	4.57	38.24	4.21	1.00	.11	**4.80**	**.55**	2.98	.34
Orthographic choice^b^	27.59	1.48	23.62	1.08	26.30	1.00	26.75	0.95	**1.00**	**.76**	0.26	.20	0.06	.04

Bold values indicate BFs and PMPs of models that received most support from the data

^a^Adjusted for educational level, language-specific didactical age and Dutch word reading

^b^Adjusted for educational level, language-specific didactical age and Dutch spelling

For French and German word reading, controlling for NL word reading ability also resulted in smaller effect sizes. The posterior means indicate that the differences in French and German word reading between averagely intelligent students with dyslexia, gifted students with dyslexia, and TD students became notably smaller, but the results are more equivocal. In both cases Model 1 received most support from the data and is now clearly favored over Model 2 (PMPs both .55). Model 1 also still received most support from the data for French orthographic choice (PMP = .64). However, the posterior means indicate that the difference in performance between averagely intelligent students with dyslexia and gifted students with dyslexia increased, which is illustrated by the smaller effect size, as averagely intelligent students with dyslexia and TD students showed comparable performance. The results for German orthographic choice also largely remained the same. The alternative model continued to receive most support from the data (PMP = .76) and the posterior means confirm the unexpected pattern of gifted students with dyslexia showing lowest performance of all groups.

## Discussion

Studies into native language (NL) skills have shown that gifted children with dyslexia outperform averagely intelligent children with dyslexia on reading and spelling tasks (e.g., Berninger & Abbott, 2013; van Viersen et al., 2016). In this study, we examined group differences between secondary school students with and without giftedness/dyslexia in foreign language (FL) reading and spelling. The first aim was to assess whether, as in the NL, the level of FL literacy of gifted students with dyslexia is higher than the literacy level of averagely intelligent students with dyslexia. On the assumption that this pattern was found, the second aim was to determine whether this difference could be accounted for by the difference in literacy level in their NL.

As a start, groups were compared on their NL literacy skills. Research in primary education has shown a stepwise pattern of group differences in NL literacy (i.e., dyslexia < gifted/dyslexia < typically developing (TD) < gifted; van Viersen et al., 2016). Finding this pattern of differences in the NL in secondary education is a precondition for the interpretation of differences in FL literacy levels. As expected, secondary school age gifted students with dyslexia were found to have higher NL word reading and spelling levels than their averagely intelligent peers with dyslexia and a full stepwise pattern of performance for NL word reading and spelling skills was attested. This result is in line with previous findings (van Viersen et al., 2016) and with the generally high stability of word reading fluency and spelling skills over time in transparent languages (e.g., Landerl & Wimmer, 2008).

The findings for FL literacy indicate that the stepwise pattern of group differences generalizes to English word reading and spelling. To test whether these group differences can be accounted for by differences in NL skills, we controlled for NL literacy, aiming to cancel out the effect of NL literacy-related subskills and NL compensatory mechanisms. As a result, effect sizes for patterns of group differences in English word reading and spelling decreased, indicating that NL skills partly influence FL performance. Nonetheless, the stepwise pattern remained present. For both word reading and spelling, the performance level of the gifted students with dyslexia approximated that of the TD students while students with dyslexia showed continuously low performance. Consequently, the higher performance of gifted students with dyslexia compared to their averagely intelligent dyslexic peers results from factors or processes that are specific to English as a FL. These findings provide support for the compensation account and are in line with studies showing that additional, language-specific factors can be responsible for higher FL literacy levels than expected based on the NL (e.g., Bekebrede et al., 2009; Morfidi et al., 2007).

These results for English literacy are interpreted in terms of compensation. The differences in literacy level between gifted and averagely intelligent students with dyslexia cannot result from less severe underlying deficits and/or higher literacy-related skills; controlling for NL literacy (and the influence of related subskills) should have cancelled out underlying group differences in the FL as well. The only way in which the higher literacy levels of the gifted students with dyslexia cannot be explained in terms of compensation is if reading and spelling in English would require an *additional* subskill. This subskill would then have to be generally impaired in students with dyslexia, but less impaired in the gifted/dyslexic students. This is unlikely, however, because multiple studies have shown that all alphabetic languages involve largely the same set of literacy-related subskills (e.g., Caravolas et al., 2012; Melby-Lervåg & Lervåg, 2011; Ziegler et al., 2010). Therefore, the current findings are considered to be in favor of the compensation account.

The stepwise pattern of group differences in NL literacy level did not generalize to French and German literacy. Gifted students with dyslexia and averagely intelligent students with dyslexia showed similar performance on the French and German literacy tasks. This did not change after controlling for NL skills. For orthographic knowledge, the absence of a stepwise pattern in French and German might be due to features of the orthographic choice task. This task did not reveal a stepwise pattern for the performance of the groups in English either. However, gifted and averagely intelligent students with dyslexia showed no differences in performance on the French and German reading tasks, while the former group clearly outperformed the latter in Dutch and English word reading. Generally, the findings for French and German as FLs align with the LCD hypothesis (Sparks & Ganschow, 1991, 1993, 1995), which proposes a strong association between the NL and FL.

There are two possible explanations for the fact that patterns of group differences in NL literacy level only generalized to English as a FL and not to French and German. The first lies in differences between the FLs in orthographic depth. German and Dutch can be considered (semi-)transparent languages whereas French is often assumed to be opaque and English even extremely opaque (Seymour et al., 2003). However, quantification of orthographic depth using entropy measures (Borgwald, Hellwig, & de Groot, 2005) indicates that French (0.46) is actually much closer to Dutch (0.23) in orthographic transparency than to English (0.83; Ziegler et al., 2010). For gifted students with dyslexia, both German (as hypothesized) and French may thus not be orthographically complex enough to evoke the development of mechanisms, as for example the processing of larger letter clusters, that help to compensate their underlying deficits and improve literacy performance (e.g., see Bekebrede et al. 2009; Morfidi et al., 2007).

A second explanation pertains to differences between the FLs in terms of exposure. In the Netherlands, both German and French are FLs that depend primarily on in-class instruction, whereas English is omnipresent in Dutch society. Although we have no data about the students’ amount of exposure to these FLs outside of the classroom, we consider it very likely that secondary school students learn to understand and use English in daily life, in contrast to French and German. This happens, for example, while watching international television shows (with Dutch subtitles), listening to popular music, or playing online computer games (see also van der Leij, Bekebrede, & Kotterink, 2010). As such, students are provided with less opportunities to develop compensatory mechanisms in French and German than in English, whereas for English children can benefit from input through multiple modalities as well as a broader language environment (see also Sparks et al., 2012).

The results of the current study have several implications. First, as it is clear that literacy outcomes may vary depending on the (characteristics of the) specific FL, future research should focus on a wider range of FLs. Also, more research on FLs that vary in the required and/or provided amount of instruction is necessary. Our findings suggest that the development of compensatory mechanisms may (partly) hinge on a combination between the orthographic depth and the amount and type of exposure of a FL. Nevertheless, more research is necessary to determine what general and language-specific factors (e.g., motivation, immersion) may influence success in FL learning. As English as a FL has a clear advantage over French and German in terms of exposure for Dutch children, it is important to consider both factors that lie in the broader language environment and lower-level (e.g., phonological processing) as well as higher-level (e.g., vocabulary, grammar) cognitive factors (Koda, 1992, 2005; Sparks & Miller, 2000). Moreover, gaining more insight in how compensatory mechanisms or strategies may influence (FL) literacy acquisition requires more advanced methods, such as eye-tracking.

Finally, it is important to note that our findings might be taken to suggest that IQ should be involved as a criterion in the diagnosis of dyslexia. For example, one might be tempted to argue that if a gifted student just misses the cut-off for low achievement, this higher literacy level could result from its high IQ, for example due to compensation. However, there are many arguments against the use of an ability-achievement discrepancy definition of dyslexia which are not questioned by the current findings (see Fletcher, Lyon, Fuchs, & Barnes, 2007 for an overview). Specifically, previous research has clearly shown that the profiles of underlying deficits associated with dyslexia are largely the same across intelligence levels. Van Viersen et al. (2015) showed that this is also valid for gifted children who just miss the cut-off for a dyslexia diagnosis based on low achievement. Their underlying profile of deficits was in accordance with their reading level. Other reasons to abandon an IQ-achievement discrepancy definition, such as that IQ is not a reliable predictor of response to remediation and does not change the effective elements of intervention (Vellutino, Scanlon, & Lyon, 2000), are not questioned either. In all, we believe that the results of the current study do not provide sufficient reason for a reinstatement of IQ as a criterion in the diagnosis of dyslexia.

Overall, this study has shown that Dutch gifted secondary school age students with dyslexia have higher NL literacy levels than their averagely intelligent dyslexic peers. As in the NL, a stepwise pattern of group differences, i.e., dyslexia < gifted/dyslexia < TD < gifted, was found for English word reading and spelling. This pattern was not found for French and German literacy performance. The higher English literacy level of gifted students with dyslexia as compared to averagely intelligent students with dyslexia results from factors or mechanisms that are unique to English as a FL. The findings suggest that gifted students with dyslexia can partly compensate for their NL dyslexia in English FL learning.

## Footnotes

1 Standardized English word reading scores based on norm categories (A = 5, E = 1) indicate the same pattern as for the raw scores at T1, Dyslexia: *M* = 2.31, *SD* = 1.31; Gifted + Dyslexia: *M* = 2.79, *SD* = 1.32; Typically Developing: *M* = 3.77, *SD* = 1.33; Gifted: *M* = 4.39, *SD* = 1.02, and T2, Dyslexia: *M* = 2.38, *SD* = 1.36; Gifted + Dyslexia: *M* = 3.40, *SD* = 1.40; Typically Developing: *M* = 4.50, *SD* = 0.73; Gifted: *M* = 4.75, *SD* = 0.68.

2 Standardized French word reading scores based on norm categories (A = 5, E = 1) indicate the same pattern as for the raw scores; Dyslexia: *M* = 1.50, *SD* = 1.10; Gifted + Dyslexia: *M* = 2.07, *SD* = 1.28; Typically Developing: *M* = 2.81, *SD* = 1.22; Gifted: *M* = 3.58, *SD* = 1.53.
